# Safety of Polyethylene Glycol Solution plus Ascorbic Acid for Bowel Preparation for Colonoscopy in Patients with Chronic Kidney Disease

**DOI:** 10.1155/2021/6696591

**Published:** 2021-03-15

**Authors:** Naoki Ohmiya, Yoshihito Nakagawa, Noriyuki Horiguchi, Takafumi Omori, Toshiaki Kamano, Kohei Funasaka, Mitsuo Nagasaka, Tomoyuki Shibata

**Affiliations:** Department of Gastroenterology, Fujita Health University School of Medicine, Toyoake, Aichi, Japan

## Abstract

**Introduction:**

Polyethylene glycol-electrolyte lavage solution plus ascorbic acid (PEG-ELS-Asc) has been recommended for colonoscopy, but little is known about the safety of PEG-ELS-Asc in patients with chronic kidney disease (CKD). The aim of this study was to determine its safety and efficacy in CKD patients.

**Methods:**

Blood and urine samples prospectively collected before and after same-day bowel preparation for colonoscopy with the conventional volume of PEG-ELS-Asc, vital signs before and after colonoscopy, and adverse events within 30 days postcolonoscopy were analyzed in consenting patients with CKD. The cleansing level was evaluated with the Boston bowel preparation score (BBPS) from colonoscopic findings.

**Results:**

Of 57 patients enrolled, 1 was excluded for refusal. Serum bicarbonate significantly dropped, and blood hemoglobin, serum total protein, albumin, aspartate aminotransferase, alanine aminotransferase, lactate dehydrogenase, total bilirubin, and uric acid significantly rose after bowel preparation, although these changes were not clinically important. Only in nondialysis patients did the platelet count and potassium significantly rise, although these changes were not clinically important either. Renal function, such as the urea, creatinine, and estimated glomerular filtration rate, was not significantly altered. An adequate bowel cleansing score, BBPS ≥ 6, was achieved in 94% of patients. The blood pressure and heart rate were not significantly different between before and after colonoscopy in either nondialysis (*n* = 32) or dialysis (*n* = 19) patients. There were no adverse events associated with bowel preparation and colonoscopy within 30 days postcolonoscopy.

**Conclusions:**

The conventional volume of same-day bowel preparation with PEG-ELS-Asc may be safe and effective in CKD patients.

## 1. Introduction

Polyethylene glycol-electrolyte lavage solution (PEG-ELS) has been a recommended cleansing agent for colonoscopy. As it is isoosmotic, PEG-ELS is considered a preferred agent in patients who are less likely to tolerate fluid intake, including patients with renal dysfunction, congestive heart failure, and cirrhosis. Use of a split-dose regimen of 4 L PEG-ELS is recommended for scheduled colonoscopy, and a same-day regimen is an acceptable alternative to split dosing, especially for patients undergoing colonoscopy in the afternoon [1]. Drinking such a large volume of PEG-ELS, however, induces poor patient compliance [2]. Another 2 L PEG-ELS agent consists of supplemental ascorbic acid and sodium sulfate but no bicarbonate. The addition of ascorbic acid and sodium L-ascorbate reduces the intake volume to only 2 L or less [3]. The cathartic effect of ascorbic acid is considered to be ascribed to its absorption mechanism, which becomes saturated at high doses [4, 5]. Excess unabsorbed ascorbic acid remains in the bowel lumen where it exerts an osmotic effect, acting synergistically with PEG. PEG-ELS plus ascorbic acid (PEG-ELS-Asc) achieved successful gut cleansing similar to 4 L PEG-ELS and better acceptability [3], but it is hyperosmotic, which can induce dehydration and electrolyte disturbance. To our knowledge, little is known about the safety of PEG-ELS-Asc in patients with chronic kidney disease (CKD) [6, 7]. In this study, we prospectively determined the safety of bowel cleansing with PEG-ELS-Asc before colonoscopy in 51 patients with CKD by comparing the blood and urine samples before and after the bowel preparation and by a 30-day postcolonoscopy chart review.

## 2. Methods

### 2.1. Study Design and Patients

This was a prospective single-center observational study of adverse events of the same-day conventional volume of PEG-ELS-Asc (MoviPrep®, EA Pharma Co., Ltd., Tokyo, Japan) by comparing the blood and urine examinations before and after bowel cleansing for conventional colonoscopy in patients with CKD. Outpatients and inpatients aged 20 years or older who were treated with CKD and required to undergo complete colonoscopy were recruited to the study between February 2015 and October 2016. The primary endpoint included clinically significant changes in urine and blood examinations including renal function. Secondary endpoints included changes in vital signs, cleansing levels at colonoscopy, and adverse events within 30 days postcolonoscopy. A sample size of 50 patients with CKD was planned to consider the risk of conventional 2 L PEG-ELS-Asc according to the previous report that same-day low-volume 1 L PEG-ELS-Asc showed significant elevation of serum creatinine concentration in 25 hemodialysis patients and 68 CKD patients without hemodialysis [6]. All patients provided written informed consent, and this study was reviewed and approved by the Institutional Review Board of Fujita Health University School of Medicine. Consenting patients were included unless they had bowel obstruction or allergy to drugs used. Women were excluded from the study if they were pregnant or at risk of becoming pregnant. Patients with phenylketonuria and glucose-6-phosphate dehydrogenase deficiency were also excluded due to the presence of aspartame and ascorbate, respectively. This study was registered with the University Hospital Medical Information Network (UMIN000013041). All methods were carried out in accordance with relevant guidelines and regulations.

### 2.2. Definition of Chronic Kidney Disease

CKD was defined as abnormalities of kidney structure or function, present for >3 months, with implications for health, and CKD was classified based on the cause and glomerular filtration rate (GFR) category [8]. GFR was estimated using the revised equations from serum creatinine for Japanese patients [9]. The estimated GFR (eGFR) was classified into 6 grades: G1: ≥90; G2: 60-89; G3a: 45-59; G3b: 30-44; G4: 15-29; and G5: <15 mL/min/1.73 m^2^. G5D corresponds to G5 undergoing dialysis.

### 2.3. Bowel Preparation

We used the same-day regimen with PEG-ELS-Asc for colonoscopy in the afternoon. Bowel preparation and colonoscopy were performed on nondialysis days in dialysis patients. On the day before examination, all patients had three meals consisting of a low-fiber diet and fasted from 9 pm, and patients with constipation took sennoside. On the day of examination, all patients drank 1,000 mL of PEG-ELS-Asc over 1-1.5 h and then 500 mL of water or clear liquid-like tea. Experienced medical staff assessed the quality of the bowel preparation by checking the clarity of patients' evacuation. When the preparation quality was poor, patients drank another 500 mL PEG-ELS-Asc for 30 min, then 250 mL of water or clear liquid. When the preparation quality was still poor, patients drank another 500 mL PEG-ELS-Asc for 30 min and then 250 mL of water or clear liquid again.

### 2.4. Measurement Parameters

Vital signs, including the blood pressure and heart rate, were recorded before and after colonoscopy on the examination table in the endoscopy center. Blood and urine were sampled before preparation and after colonoscopy within two hours. Urine was sampled from all patients except on dialysis without urination. The measured parameters of blood and urine samples are shown in Table 1.

### 2.5. Colonoscopy Procedure

A conventional colonoscope (CF-HQ290I, PCF-Q260AZI, and PCF-H290I, Olympus Corporation, Tokyo, Japan; EC-L590ZW, Fujifilm Co., Ltd., Tokyo, Japan) was used for all colonoscopic examinations. When colorectal polyps or tumors were observed, endoscopic resection including polypectomy and endoscopic mucosal resection (EMR) was performed if possible without hospitalization. Most procedures were performed with air insufflation but CO_2_ insufflation in some cases. Conscious sedation was performed, mostly with midazolam (2–10 mg), according to the patient's request or pain status in previous procedures. Antispasmodics such as scopolamine butylbromide or glucagon were administered depending upon the colonoscopist's decision. Monitoring with an automatic blood pressure monitor was performed in all cases during sedation.

### 2.6. Cleansing Levels at Colonoscopy

The cleansing level was evaluated in the right colon, transverse colon, and left colon with the Boston bowel preparation score (BBPS) from colonoscopic findings [10]. The total score of the BBPS was categorized into two groups: 6–9 and 0–5. BBPS ≥ 6 was defined as good preparation, and BBPS 0–5 was defined as poor preparation.

### 2.7. Statistical Analysis

The numbers in the text are expressed as the median and range or mean and standard deviation, depending upon the fit of a normal distribution. The numbers were tested for normality using the D'Agostino test, the Shapiro-Wilk test, and the Kolmogorov-Smirnov test. Comparisons were analyzed between the variables before and after bowel preparation or colonoscopy using the Wilcoxon signed rank test or the paired *t*-test. When the cohort population was less than 30, the Wilcoxon signed rank test was used. Differences were considered significant with *P* values less than 0.05.

## 3. Results

### 3.1. Patients with Chronic Kidney Disease

Of 57 patients with CKD enrolled between February 2015 and October 2016, 1 was excluded because he refused bowel preparation and colonoscopy.  Table 2 shows the clinical characteristics of the remaining 56 patients. Five patients did not undergo blood examination after colonoscopy. Regarding drugs associated with hypokalemia, diuretics and polystyrene cation-exchange resin were used in 32 (57%) and 3 (5%) patients, respectively. Of 32 patients on these drugs, 3 took diuretics and the remaining 29 discontinued the drugs on the day of colonoscopy.

### 3.2. Colonoscopy

Of 56 patients, 27 underwent colonoscopy without biopsy or endoscopic treatment, 5 underwent colonoscopy with biopsy, and 24 underwent colonoscopy with endoscopic polyp resection.  Table 3 shows the volume of bowel preparation and Boston bowel preparation score.

### 3.3. Measurement Parameters

#### 3.3.1. Blood Samples

Tables 4 and 5 show the blood parameters before and after bowel preparation in all (*n* = 51), nondialysis (*n* = 32), and dialysis patients (*n* = 19). In both the nondialysis and dialysis patients, bicarbonate significantly dropped, and hemoglobin, total protein, albumin, aspartate aminotransferase (AST), alanine aminotransferase (ALT), lactate dehydrogenase (LD), total bilirubin, and uric acid significantly rose, although these changes were clinically insignificant. Only in nondialysis patients did the platelet count and potassium significantly rise, although these changes were also clinically insignificant. Renal function, such as urea, creatinine, and eGFR, was not significantly changed either in nondialysis patients or in dialysis patients.

In 35 patients without the use of sedatives or antispasmodics during colonoscopy, AST, ALT, LD, and total bilirubin were significantly changed: 15 to 19 IU/L, 11 to 14 IU/L, 200 to 215 IU/L, and 0.5 to 0.6 mg/dL (median, *P* < 0.001, <0.001, <0.001, and 0.002), respectively. In 16 patients with the use of sedatives or antispasmodics during colonoscopy, AST and ALT were significantly changed: 17.5 to 19.5 IU/L and 10 to 12 IU/L (median, *P* = 0.004 and 0.022), respectively, while changes in LD and total bilirubin were not significant (median, 208 to 224 IU/L and 0.5 to 0.5 mg/dL, *P* = 0.061 and 0.244, respectively). In 29 patients who underwent colonoscopy without any endoscopic intervention except biopsy, AST, ALT, LD, and total bilirubin were significantly changed: 17 to 20 IU/L, 10 to 13 IU/L, 207 to 226 IU/L, and 0.5 to 0.6 mg/dL (median, *P* < 0.001, <0.001, <0.001, and 0.007), respectively. In 22 patients who underwent colonoscopy with endoscopic intervention, AST and ALT were significantly changed: 15 to 18.5 IU/L and 11 to 13.5 IU/L (median, *P* = 0.002 and 0.010), respectively, while changes in LD and total bilirubin were not significant (median, 198 to 210 IU/L and 0.5 to 0.5 mg/dL, *P* = 0.251 and 0.064, respectively).

In a male nondialysis patient with nephrotic syndrome due to membranous nephropathy, transient liver injury developed: AST: 27 to 226 IU/L; ALT: 29 to 153; LD: 200 to 394; and total bilirubin: 0.3 to 0.4. He had been treated with 30 mg of prednisolone for 7 days before colonoscopy with endoscopic resection of three polyps in addition to prophylactic administration of trimethoprim-sulfamethoxazole starting in the morning of the examination day. During colonoscopy, he took no medications including sedatives or antispasmodics. Even though trimethoprim-sulfamethoxazole was continued thereafter, liver injury was spontaneously recovered as follows: AST: 64 and 17; ALT: 101 and 37; and LD: 218 and 194 on postcolonoscopy days 1 and 4, respectively, which may have been ascribed to PEG-ELS-Asc.

Serum potassium levels significantly increased after bowel preparation only in nondialysis patients but not in dialysis patients. Serum potassium levels tended to increase in nondialysis patients on diuretics and not on diuretics (median, 4.25 to 4.50 mEq/L and 4.50 to 4.60 mEq/L; *P* = 0.134 and 0.069, respectively). Regarding the intake volume of PEG-ELS-Asc, median serum potassium levels before and after were 4.3 and 4.2 mEq/L in patients on 1.5 L or more, respectively (*P* = 0.566), and 4.95 to 4.85 in patients on less than 1.5 L, respectively (*P* = 0.431).

#### 3.3.2. Urine Samples

Table 4 shows the urine parameters before and after bowel preparation in all nondialysis and dialysis patients who could provide paired urine samples. Of 32 nondialysis and 19 dialysis patients, 30 (94%) and 11 (58%) provided urine samples, respectively. In nondialysis patients, urine potassium and osmolarity were significantly higher and urine chloride was significantly lower after bowel preparation with PEG-ELS-Asc than before. In dialysis patients, urine potassium was significantly higher after bowel preparation with PEG-ELS-Asc than before.

#### 3.3.3. Vital Signs

Supplementary Table shows the blood pressure and heart rate before and after colonoscopy in all (*n* = 51), nondialysis (*n* = 32), and dialysis patients (*n* = 19). The systolic and diastolic blood pressure and heart rate did not significantly differ after colonoscopy from before colonoscopy in either nondialysis or dialysis patients.

#### 3.3.4. Adverse Events within 30 Days Postcolonoscopy

Blood was sampled in 35 patients (69%) within 30 days postcolonoscopy. Three patients developed aspiration pneumonia on the 12^th^ day (CKD stage G5A3), transient ischemic attack on the 20^th^ day (CKD stage G3aA3), and pulmonary edema on the 29^th^ day (CKD stage G5A3) postcolonoscopy, respectively. No nondialysis patients developed into a dialysis stage. There were no adverse events associated with bowel preparation and colonoscopy.

## 4. Discussion

The safety and usefulness of PEG-ELS have been described in many studies. The present study demonstrated that a conventional volume of PEG-ELS-Asc was safe for patients with CKD whether on dialysis or not. Yoshida et al. reported that a same-day low volume of 1 L PEG-ELS-Asc and 0.5 L water was safe in elderly patients aged ≥80 years and patients with renal dysfunction in a single-center retrospective analysis, in which there were significant differences in blood hematocrit (36.7 ± 6.1% vs. 39.0 ± 5.7%) and serum creatinine levels (1.30 ± 0.12 mg/dL vs. 1.37 ± 0.14 mg/dL) before and after bowel preparation in 68 renal dysfunction patients without hemodialysis (serum creatinine level, 1.1-2.9 mg/mL), while there were no significant differences except for serum creatinine levels (6.14 ± 1.2 mg/dL vs. 6.80 ± 1.4 mg/dL) in 25 hemodialysis patients. Lee et al. reported that the laboratory findings were not significantly different before and after the administration of 2 L polyethylene glycol plus ascorbic acid or 4 L polyethylene glycol. In both groups, the estimated glomerular filtration rate was not influenced by the administration of the bowel cleansing agent [7]. The present study using a same-day regimen of the conventional volume of PEG-ELS-Asc (median volume 1.5 L of PEG-ELS-Asc and 750 mL of water or clear liquid) demonstrated that blood hemoglobin, serum total protein, albumin, AST, ALT, LD, and total bilirubin were significantly increased, and bicarbonate was significantly decreased after preparation in both the nondialysis and dialysis patients. The blood platelet count, serum potassium, and uric acid levels were increased only in nondialysis patients, while renal function represented by blood urea nitrogen, serum creatinine, and eGFR was not significantly worsened after bowel preparation in either nondialysis or dialysis patients. These elevated blood hemoglobin, serum total protein, and albumin levels were observed even in patients without comorbidities due to dehydration and hemoconcentration [11, 12] and showed subtle changes; therefore, they seemed clinically irrelevant. A significant decrease in serum sodium bicarbonate after the intake of PEG-ELS-Asc was also observed in patients without comorbidities [11]. PEG-ELS-Asc (MoviPrep) contains 11.8 g (59.6 mmol) of sodium L-ascorbate per pack as an alkaline agent instead of bicarbonate contained in PEG-ELS because the combination of ascorbic acid and bicarbonate releases carbon dioxide as a byproduct of the acid-base reaction, and 2 L of PEG-ELS-Asc stimulates 3 L of defecation accompanied by the loss of bicarbonate. This significant decrease in serum sodium bicarbonate, however, seems clinically irrelevant because of bicarbonate and an electrolyte-water balance parameter; serum osmolarity remained within the normal ranges after colonoscopy in most patients with CKD. A significant increase in AST, ALT, and LD was observed after colonoscopy. Elevated ALT levels were also observed even in patients without comorbidities using both PEG-ELS and PEG-ELS-Asc [11]. These affected hepatic parameters were irrelevant to the use of sedatives and antispasmodics during colonoscopy or endoscopic intervention in the present study. Although these changes remained within the normal ranges in most patients, one patient showed transient abnormal liver function. The mechanism of altered hepatic parameters is still unknown, but it may have been due to transient hypoperfusion. An increase in total bilirubin is considered due to a circadian rhythm or fasting for a long time on an examination day [13]. The important finding of the present study is renal function represented by serum BUN, creatinine, and eGFR, and significant electrolyte disturbance was not observed after bowel preparation, and nondialysis patients did not develop into a dialysis stage. A slight increase in serum potassium and uric acid observed only in nondialysis patients presumably due to dehydration is considered clinically irrelevant. This increase in serum potassium was not associated with the disuse of diuretics or the intake volume of PEG-ELS-Asc. The reason why serum potassium increased only in nondialysis patients is unknown. The urine potassium level was significantly increased in both the nondialysis and dialysis patients, and urine chloride was significantly decreased, and osmolarity was significantly increased only in nondialysis patients, which was considered to compensate for blood changes. The efficacy of the conventional volume of PEG-ELS-Asc in CKD patients was satisfactory because an adequate bowel cleansing score was achieved in 94% of patients in the present study, which was equivalent to the previous studies in subjects without comorbidities [3, 14].

Recently, a 1 L PEG-ELS-Asc solution (Plenvu®; Norgine, Harefield, United Kingdom) has been introduced on the market, and it was reported to allow overall cleansing success, high-quality cleansing of the right colon, and tolerability, compared to sodium picosulfate with magnesium citrate [15], 4 L PEG preparation, and 2 L PEG-ELS-Asc solution [16]. Plenvu® consists of as much as 48.11 grams of sodium L-ascorbate but doses of other elements similar to MoviPrep®. Therefore, Plenvu® may also be safe in patients with CKD.

The present study has inherent limitations, including a small sample size of unconsecutive patients, a single-center study without controls, and blood and urine analysis after colonoscopy. Further studies in a larger population are required to confirm our findings. In conclusion, our study suggests the safety and efficacy of bowel preparation with a conventional volume of PEG-ELS-Asc for colonoscopy in CKD patients regardless of hemodialysis. Blood and urine examinations before and after colonoscopy may not be routinely monitored when using PEG-ELS-Asc solution (MoviPrep®).

## Figures and Tables

**Table 1:** Measurement parameters before and after bowel preparation for colonoscopy.

Vital signs	Blood pressure, heart rate
Urine examination	Na, K, Cr, RBC, WBC, osmotic pressure
Blood examination	CBC, Na, K, Cl, Ca, P, Mg, BUN, Cr, eGFR, TP, Alb, AST, ALT, LD, TBil, UA, HCO_3_^−^, osmolarity

Na: sodium; K: potassium; Cr: creatinine; RBC: red blood cell: WBC: white blood cell; CBC: complete blood count; Ca: calcium; P: phosphate; Mg: magnesium; BUN: blood urea nitrogen; eGFR: estimated glomerular filtration rate; TP: total protein; Alb: albumin; AST: aspartate aminotransferase; ALT: alanine aminotransferase; LD: lactate dehydrogenase; TBil: total bilirubin; DBil: direct bilirubin; CRP: C-reactive protein; UA: uric acid; HCO_3-_: bicarbonate.

**Table 2:** Clinical characteristics of patients with chronic kidney disease.

Characteristics	Total	%
No.	56	
Age (years)		
Mean	65	
Standard deviation	12	
Sex		
Male	38	68%
Female	18	32%
Healthcare services		
Outpatient	32	57%
Inpatient	24	43%
Height (cm)		
Mean	159.6	
Standard deviation	9.0	
Body weight (kg)		
Mean	59.5	
Standard deviation	13.5	
Body mass index		
Mean	23.2	
Standard deviation	4	
Cause of chronic kidney disease		
Diabetic nephropathy	20	36%
Hypertensive nephrosclerosis	16	29%
Membranous nephropathy	4	7%
Polycystic kidney	2	4%
Tubulointerstitial nephritis	2	4%
Minimal change nephrotic syndrome	1	2%
IgA nephropathy	1	2%
Sarcoidosis-related tubulointerstitial nephritis	1	2%
Lupus nephritis	1	2%
Fanconi syndrome	1	2%
Ischemic nephropathy	1	2%
Reflux nephropathy	1	2%
Postnephrectomy and chemotherapy for renal pelvis cancer	1	2%
Kidney transplant recipient	1	2%
Miscellaneous	3	5%
Classification of chronic kidney disease		
G2 (eGFR: 60-89)	1	2%
G3a (eGFR: 45-59)	4	7%
G3b (eGFR: 30-44)	10	18%
G4 (eGFR: 15-29)	6	11%
G5 (eGFR: <15)	14	25%
G5D (eGFR: <15, dialysis)	21	38%
Indications		
FIT positive	24	43%
Melena or hematochezia	6	11%
Follow-up	6	11%
Anemia	3	5%
Diarrhea or constipation	4	7%
Abdominal pain	1	2%
Other screening^∗^	12	21%
Medication		
Diuretics	32	57%
Polystyrene cation-exchange resin	3	5%
Previous laparotomy		
Presence	11	22%
Absence	45	88%

eGFR: estimated glomerular filtration rate; FIT: fecal immunological test.

**Table 3:** Bowel preparation and colonoscopy.

Variables	Total (%)
Polyethylene glycol-electrolyte lavage solution plus ascorbic acid (mL)	
Mean	1,496
Standard deviation	381
Water or clear liquid (mL)		
Median	750
Range	0-2,000
Time from the beginning of bowel preparation to clear evacuation (min)	
Median	182.5
Range	50-542
Boston bowel preparation score	
Total score	
Median	8
Range	2-9
Good preparation (6-9)	52 (93%)
Poor preparation (0-5)	4 (7%)
Right colon	
Median	2
Range	0-3
Transverse colon	
Median	3
Range	1-3
Left colon	
Median	3
Range	1-3

**Table 4:** Comparison of blood and urine parameters before and after bowel preparation with polyethylene glycol solution plus ascorbic acid in all patients.

Parameter	Normal range	All patients (*n* = 51)		
		Before preparation	After preparation	*P*
Blood samples				
White blood cell count (/*μ*L)	3,300-8,600	5,800 (2,900-16,100)	6,800 (2,900-25,600)	0.035
Hemoglobin (g/dL)	13.7-16.8 (male)	11.0 ± 1.5	11.4 ± 1.5	0.001
	11.6-14.8 (female)			
Platelet count (×10^4^/mL)	15.8-34.8	20.6 (11.1-49.7)	21.2 (11.7-45.7)	0.015
Na (mEq/L)	138-146	140 ± 3.7	141 ± 3.5	0.060
K (mEq/L)	3.6-4.9	4.5 ± 0.7	4.5 ± 0.7	0.088
Cl (mEq/L)	99-109	103 ± 4.8	104 ± 4.4	0.076
Ca (mg/dL)	8.7-10.3	9.2 ± 0.8	9.3 ± 0.7	0.217
P (mg/dL)	2.5-4.7	4.1 (2.7-7.5)	4.4 (2.2-9.2)	0.068
Mg (mg/dL)	1.8-2.4	2.0 (1.1-3.1)	2.0 (1.1-3.4)	0.341
BUN (mg/dL)	8.0-22.0	35.8 (8.3-95.4)	30.0 (7.5-96.1)	0.431
Cr (mg/dL)	0.6-1.1	4.68 ± 3.06	4.89 ± 3.23	0.075
eGFR (mL/min/1.73m^2^)	≥60.0	10.1 (3.8-76.4)	9.5 (3.5-84.2)	0.376
TP (g/dL)	6.7-8.3	6.8 (3.9-8.1)	7.3 (4.7-8.3)	<0.001
Alb (g/dL)	4.0-5.0	4.0 (1.4-5.0)	4.2 (1.8-5.3)	<0.001
AST (IU/L)	13-33	16 (3-50)	19 (4-226)	<0.001
ALT (IU/L)	6-30	11 (2-64)	13 (51-153)	<0.001
LD (IU/L)	119-229	205 (151-338)	222 (132-439)	<0.001
TBil (mg/dL)	0.3-1.2	0.5 (0.2-1.2)	0.5 (0.2-1.3)	0.001
UA (mg/dL)	3.6-7.0	5.8 ± 1.5	6.2 ± 1.5	0.002
HCO_3_^−^ (mmol/L)	20.0-30.0	25.6 ± 3.4	23.3 ± 3.3	<0.001
Osmolarity (mOsm/kgH_2_O)	275-290	294 (267-317)	295 (265-313)	0.906
Urine samples				
Na (mEq/L)	ND	78 (16-147)	69 (6-190)	0.882
K (mEq/L)	ND	22 (4-71)	36 (4-91)	<0.001
Cl (mEq/L)	ND	71 (10-151)	52 (10-138)	0.030
Osmolarity (mOsm/kgH_2_O)	50-1300	307 (126-691)	346 (114-752)	0.007

Median (range); mean ± standard deviation. ND: not determined; WBC: white blood cell; Na: sodium; K: potassium; Ca: calcium; P: phosphate; Mg: magnesium; BUN: blood urea nitrogen; Cr: creatinine; eGFR: estimated glomerular filtration rate; TP: total protein; Alb: albumin; AST: aspartate aminotransferase; ALT: alanine aminotransferase; LD: lactate dehydrogenase; TBil: total bilirubin; DBil: direct bilirubin; CRP: C-reactive protein; UA: uric acid; HCO_3_^−^: bicarbonate.

**Table 5:** Comparison of blood and urine parameters before and after bowel preparation with polyethylene glycol solution plus ascorbic acid in nondialysis and dialysis patients.

Parameter	Normal range	Nondialysis patients (*n* = 32)			Dialysis patients (*n* = 19)		
		Before preparation	After preparation	*P*	Before preparation	After preparation	*P*
Blood samples							
White blood cell count (/*μ*L)	3,300-8,600	6,450 (3,700-16,100)	7,250 (2,900-13,000)	0.098	5,700 (2,900-15,800)	6,200 (3,000-25,600)	0.219
Hemoglobin (g/dL)	13.7-16.8 (male)	11.2 (7.2-13.2)	11.5 (8.6-14.8)	0.042	10.8 (8.4-14.4)	11.4 (9.4-14.8)	0.004
	11.6-14.8 (female)						
Platelet count (×10^4^/mL)	15.8-34.8	20.5 (11.1-36.9)	21.1 (12.1-42.1)	0.046	20.7 (12.4-49.7)	21.2 (11.7-45.7)	0.177
Na (mEq/L)	138-146	141 ± 4	141 ± 4	0.204	138 (132-144)	139 (135-144)	0.134
K (mEq/L)	3.6-4.9	4.3 (2.8-5.5)	4.5 (2.9-5.5)	0.022	4.7 (3.2-6.0)	4.7 (3.1-6.1)	0.160
Cl (mEq/L)	99-109	106 (94-113)	107 (97-112)	0.060	100 (89-108)	100 (96-104)	0.482
Ca (mg/dL)	8.7-10.3	9.0 ± 0.7	9.1 ± 0.7	0.357	9.4 (8.0-12.0)	9.5 (8.5-11.4)	0.407
P (mg/dL)	2.5-4.7	3.7 (2.7-6.7)	3.9 (2.2-9.2)	0.231	4.7 (3.4-7.5)	5.1 (3.2-8.0)	0.190
Mg (mg/dL)	1.8-2.4	2.0 (1.1-2.5)	1.9 (1.1-3.4)	0.104	2.2 (1.7-3.1)	2.2 (1.7-3.1)	0.780
BUN (mg/dL)	8.0-22.0	28.0 (8.3-95.4)	33.8 (7.5-96.1)	0.098	36.1 (20.3-59.8)	30.0 (19.2-60.7)	0.573
Cr (mg/dL)	0.6-1.1	2.13 (0.59-8.16)	2.13 (0.54-8.59)	0.108	7.06 (2.88-12.50)	7.75 (2.21-12.62)	0.108
eGFR (mL/min/1.73m^2^)	≥60.0	25.5 (6.0-76.4)	24.5 (5.7-84.2)	0.510	5.5 (3.8-20.3)	6.0 (3.5-20.2)	0.519
TP (g/dL)	6.7-8.3	6.7 (3.9-8.1)	7.2 (1.44.8-8.1)	<0.001	7.1 (4.0-7.9)	7.3 (4.7-8.3)	0.006
Alb (g/dL)	4.0-5.0	3.9 (1.4-4.8)	4.0 (1.8-5.3)	<0.001	4.0 (1.9-5.0)	4.3 (2.1-4.8)	0.008
AST (IU/L)	13-33	18 (6-50)	23 (7-226)	<0.001	13 (3-24)	16 (4-29)	0.001
ALT (IU/L)	6-30	11 (6-64)	14 (5-153)	<0.001	10 (2-25)	13 (2-26)	<0.001
LD (IU/L)	119-229	208 (159-338)	226 (132-439)	0.006	177 (151-249)	197 (158-284)	0.004
TBil (mg/dL)	0.3-1.2	0.5 (0.2-1.0)	0.6 (0-1.1)	0.011	0.5 (0.2-1.2)	0.5 (0.2-1.3)	0.021
UA (mg/dL)	3.6-7.0	6.2 ± 1.4	6.6 ± 1.3	<0.001	5.3 (2.9-7.6)	5.0 (3.6-8.5)	0.107
HCO_3_^−^ (mmol/L)	20.0-30.0	25.1 ± 3.6	22.8 ± 3.4	<0.001	26.1 (22.5-31.1)	24.1 (19.8-28.6)	0.005
Osmolarity (mOsm/kgH_2_O)	275-290	297 ± 12	297 ± 11	0.749	295 (280-315)	292 (282-311)	0.556
Urine samples							
Na (mEq/L)	ND	71 (16-147)	65 (16-190)	0.689	90 (50-127)	77 (31-126)	0.756
K (mEq/L)	ND	26 (6-71)	37 (11-91)	<0.001	16 (4-42)	21 (4-69)	0.021
Cl (mEq/L)	ND	72 ± 40	58 ± 32	0.029	83 (41-118)	66 (18-113)	0.594
Osmolarity (mOsm/kgH_2_O)	50-1300	329 (126-691)	360 (120-752)	0.017	279 (186-328)	287 (114-343)	0.110

Median (range); mean ± standard deviation. ND: not determined; WBC: white blood cell; Na: sodium; K: potassium; Ca: calcium; P: phosphate; Mg: magnesium; BUN: blood urea nitrogen; Cr: creatinine; eGFR: estimated glomerular filtration rate; TP: total protein; Alb: albumin; AST: aspartate aminotransferase; ALT: alanine aminotransferase; LD: lactate dehydrogenase; TBil: total bilirubin; DBil: direct bilirubin; CRP: C-reactive protein; UA: uric acid; HCO_3_^−^: bicarbonate.

## Data Availability

The data used to support the findings of this study are included within the article.

## Abbreviations

PEG-Asc: Polyethylene glycol plus ascorbate
CKD: Chronic kidney disease.

## Supplementary Materials

Supplementary Materials Supplementary Table: vital signs before and after 56 cases.
